# Rethinking postdoc careers through the science of science

**DOI:** 10.1073/pnas.2500344122

**Published:** 2025-02-24

**Authors:** Wu Youyou, Kaiyun Feng

**Affiliations:** ^a^Department of Psychology and Human Development, Institute of Education, University College London, London WC1H 0AH, United Kingdom; ^b^King’s Academy, King’s College London, London WC2R 1AE, United Kingdom; ^c^Student Admissions and Access, University of Cambridge, Cambridge CB2 3PT, United Kingdom

Postdocs are referred to as the invisible scholars (1). Their position in academia is ambiguous and overlooked, except perhaps when a university aims to inflate its number of Nobel laureates by claiming former postdoctoral researchers.

The invisibility of postdocs is unfortunately mirrored in the field of Science of Science, a field situated at the intersection of data science and the study of the scientific enterprise. The field’s key goals include “enhancing career paths for scientists” as outlined in their landmark review paper. While it has made notable progress in mapping out career dynamics [see Wang and Barabási’s book for a review (2)], the study of postdocs as a career stage has yet to catch the wave of the quantitative Science of Science approach.

Duan et al.’s work (3) is one of the few attempts to characterize a successful postdoc journey using the data-driven approach. Based on a dataset of ~45 k individuals, the project investigates the impact of postdoctoral training on securing a faculty position and achieving initial success.

As it turns out, postdoc life is not just about surviving on free pizzas at seminars—it is also about producing strong publications. Publishing a high-impact paper during the postdoc phase boosts the likelihood of securing a faculty position from below 60% to above 65%. This holds true even if an individual has already published a strong paper during their PhD. Notably, this fits with Liénard et al.’s previous finding that the success and experience of an individual’s postdoc mentor matters even more than that of their PhD mentor (4).

The study also found that a moderate shift in research topics from PhD to postdoc enhances initial career success compared to no change (*d* = 0.54). This reinforces Liénard et al.’s insight that having PhD and postdoc mentors with highly similar expertise can actually hinder career growth, regardless of the mentors’ track record (4).

Finally, the study examined the impact of mobility and found that moving to a different country for a postdoc positively influences initial career success (*d* = 0.14), even for American PhDs. This again aligns with Liénard et al.’s finding that having “out-of-nest” mentors is beneficial (4). On the other hand, the current study found no advantage in moving to a prestigious institution for a postdoc (*d* < 0.01), despite many researchers aiming to “level up.” However, their study focused only on moves to the top 10 universities, so the broader effects of institutional prestige remain uncertain.

Connecting these findings, the postdoc phase emerges as a new stage where prior achievements take a backseat and fresh accomplishments under the guidance of a strong mentor come to the forefront. Both diversity in research topics and social networks are valuable.

Why have postdocs not been the focus in Science of Science? One reason is the lack of large comprehensive datasets. The Mathematics Genealogy Project and Academic Family Tree are the main sources that track postdocs. The former focuses exclusively on mathematicians, and the latter predominantly tracks neuroscientists. In the current study, the authors used data from a professional networking site spanning 19 disciplines, offering a potentially more balanced perspective. Future studies could leverage these data to integrate postdocs into the broader investigation of scientific careers. Through this new lens, scholars can revisit familiar topics like productivity, mobility, structural biases, mentorship, and collaboration.

Returning to the practical implications of the current study, Duan et al.’s work provides newly minted PhDs with a roadmap to maximize their postdoc experience—highlighting possibilities to pivot research directions, build new connections, and pursue international experiences. These insights challenge the notion that pursuing a postdoc position has to be the last resort. When approached strategically, the postdoc phase can serve as a powerful springboard for long-term career advancement (see Heggeness et al.’s study for a concrete example ref. 5).

On a broader scale, informed choices made to optimize individual postdoc journeys can also collectively advance global scientific progress. For institutions and countries, attracting talents from around the world strengthens their research ecosystems (6). Supporting researchers in interdisciplinary collaboration can drive innovation and knowledge production (7).

However, these opportunities do not come gift-wrapped—they are loaded with challenges and pitfalls that can spiral into the infamous postdoc depression (8). To encourage individuals to pursue these paths, robust policies must be in place at institutional and national levels to provide adequate support. Below, we outline a few factors influencing postdoc experiences, each of which requires further research, evaluation, and implementation.

- Reliable immigration pathways: A key obstacle to international mobility is securing stable residency or permanent status. A stable legal status provides the freedom to focus on research and career advancement without bureaucratic hurdles. Many countries have implemented special accelerated visa and residency programs tailored for researchers, such as the UK’s Global Talent Visa, France’s Passeport Talent, and European Union’s Blue Card. A quick and predictable immigration pathway is notoriously lacking for researchers in the United States, especially for researchers from countries like China and India, where strict caps based on country of birth significantly limit career prospects.
- The visibility of international positions: Most researchers are unfamiliar with job positions and recruitment processes in other countries. Organizational efforts to actively reach potential researchers outside their own borders can enhance international mobility. Examples include international job portals like EURASESS and Management PhD job doc, international fellowship programs like Marie Skłodowska-Curie Actions and Fulbright Scholar Program, and professional development sessions at international conferences and societies.
- Job security: Due to the temporary and transitional nature, postdoc positions around the world often have little job security. To improve this circumstance, more institutional support is needed. Examples include creating permanent research associate positions or faculty-track postdoc positions. Besides, diversifying career paths beyond academia can also offer postdocs more stable employment, as nonacademic roles often provide permanent contracts and better career prospects.
- Financial support: postdocs are known to be the “cheap labor.” More recently, the NIH increased the minimum salary for postdoctoral researchers to $61,008, which, while an improvement, still lags behind industry standards. Beyond improving postdoctoral researchers’ salaries, potential avenues for increasing financial support include tax benefits, unemployment insurance, and housing benefits. Implementing these solutions requires institutional effort as well as recognition of their value by governments and funding bodies, necessitating a long-term commitment.
- Structural disadvantage: The combination of low income, minimal job security, and international relocation is a recipe for mental struggles, discouraging many from continuing their postdoc position. These challenges disproportionately affect certain groups. For instance, female academics face immense obstacles in balancing motherhood with career progression, resulting in higher attrition rates at the postdoc stage (9). Addressing these systemic issues requires comprehensive reforms, including equitable compensation, enhanced job security, and family-friendly policies.

“Duan et al.’s work (3) is one of the few attempts to characterize a successful postdoc journey using the data-driven approach.”

The postdoc opportunities highlighted in Duan and colleagues’ work (3) resonate deeply with our personal experiences. One of us moved to a different country and the other to a new city for our postdoc positions. The interdisciplinary exposure, collaboration networks, and institutional reputation proved invaluable, continuing to benefit our careers to this day. Yet, the challenges were just as real—putting personal life on hold, adapting to a new environment, and navigating the complexities of visa processes. Through it all, we hoped to be seen as more than just a fleeting presence in the system. As postdocs, we wanted to be recognized as valued contributors to the lab, the university, and the country—not just if we can win a Nobel Prize.
